# Addendum: Data splitting against information leakage with DataSAIL

**DOI:** 10.1038/s41467-025-67495-w

**Published:** 2026-02-13

**Authors:** Roman Joeres, David B. Blumenthal, Olga V. Kalinina

**Affiliations:** 1 Helmholtz Institute for Pharmaceutical Research Saarland (HIPS), Helmholtz Centre for Infection Research (HZI), Saarbrücken, Germany; 2 Center for Bioinformatics, Saarland University, Saarbruücken, Germany; 3 Department Artificial Intelligence in Biomedical Engineering, Friedrich-Alexander-Universität Erlangen-Nürnberg, Erlangen, Germany; 4 Medical Faculty, Saarland University, Homburg, Germany

Addendum to: *Nature Communications* 10.1038/s41467-025-58606-8, published online 8 April 2025

In our recently published manuscript, we presented the DataSAIL tool for automatically splitting datasets used in the training of machine learning models while minimizing information leakage between the splits (e.g., training, validation, and test sets). In evaluating our method, we utilized several benchmark datasets and compared DataSAIL to existing tools and splitting algorithms. While the publication focused on comparing automated tools for data splitting, many of the datasets used in our study have been published together with corresponding data splits created by the authors. In this addendum, we extend our analysis to comparing DataSAIL splits to these predefined data splits. We achieve this by measuring the amount of leaked information between splits using the scaled version of the leakage score *L*(*π*), as defined in Eqs. (2) and (20) of our original article. Apart from the three datasets used in our paper—MoleculeNet^1^, LP-PDBBind^2^, and PLINDER^3^—we also extend our analysis to PINDER^4^ and the human gold-standard dataset for protein-protein interaction (PPI) prediction by Bernett et al.^5^. This is done for completeness and due to their popularity in the field of PPI prediction.

## MoleculeNet

The authors of MoleculeNet provide recommended splits for each dataset. In Table 1, they are computed using the DeepChem package^6^ and primarily comprise a random data split. Exceptions include QM7, which features a stratified split, and HIV, BACE, and BBBP, which have scaffold splits. Each dataset is split into 80% training data, 10% validation data, and 10% test data. In our original work, we utilized DataSAIL’s similarity-based splitting to achieve an 80:20 separation between the training and test data. Therefore, we had to redo the data splitting, leading to slightly different leakage measurements compared with Table 1 in the original publication. In all cases, DataSAIL splits offer reduced data leakage.

**Table 1:** DataSAIL on MoleculeNet

	MoleculeNet		DataSAIL S1
Dataset	recomm. technique	Scaled *L*(*π*)	Scaled *L*(*π*)
QM7	stratified	0.3425	**0.2680**
QM8	random	0.3300	**0.2918**
QM9	random	0.3306	**0.2727**
ESOL	random	0.3069	**0.1808**
Freesolv	random	0.3231	**0.1410**
Lipophilicyty	random	0.3343	**0.3027**
MUV	scaffold	0.3349	**0.3143**
HIV	scaffold	0.3306	**0.3071**
BACE	random	0.3309	**0.3036**
BBBP	scaffold	0.3366	**0.2866**
Tox21	random	0.3333	**0.2224**
ToxCast	random	0.3355	**0.2220**
SIDER	random	0.3513	**0.2345**
ClinTox	random	0.3317	**0.2303**

Comparison of the scaled *L*(*π*) of the recommended splits by MoleculeNet to the DataSAIL S1 split. The minimal data leakage is highlighted in **bold**.

## LP-PDBBind

The authors of the Leak-Proof PDBBind (LP-PDBBind) dataset^2^ also provide a data split that reduces data leakage between the training, validation, and test sets (Table 2). Their approach guarantees a maximum protein sequence identity of 50% between the training split and any other split, and a maximum protein sequence identity of 90% between the validation and test data. Furthermore, the maximum ligand similarity between any two splits is 0.99. The protein similarity is computed as the percentage of matching residues after a Needleman-Wunsch alignment, while the ligand similarity is measured as the Dice similarity between Morgan Fingerprints.

**Table 2:** DataSAIL on LP-PDBBind

Split Method	Scaled *L*(*π*)
LP-PDBBind	0.4484
DataSAIL Ligand S1	0.6330
DataSAIL Protein S1	0.5446
DataSAIL S2	**0.4277**

Comparison of the scaled *L*(*π*) of the provided split by Li et al. to three DataSAIL splits. The minimal data leakage is highlighted in **bold**.

The approach of providing maximum similarity limits between splits is different from DataSAIL’s approach of minimizing the total amount of leaked information, which we also describe in the section “The (*k*, *R*, *C*)-DataSAIL problem” and Eq. (1) in the original publication. While Eq. (1) is derived from a different publication (Elangovan et al.^7^), it describes the concept of minimizing the maximum single leak. As LP-PDBBind provides a split into approximately 61% training data, roughly 26% validation data, and approximately 13% test data, we had to recalculate the DataSAIL split here as well. This explains the deviation of the values reported here from the main manuscript. The DataSAIL S2 split offers the smallest data leakage, despite being generated by an automated procedure, whereas the LP-PDBBind split was explicitly designed for this task.

## PLINDER

The Protein Ligand INteraction Dataset and Evaluation Resource (PLINDER) is a dataset containing protein-ligand interactions extracted from the PDB. The authors provide three splits, among which the PLINDER-PL50 is the most complex. It combines four similarity metrics for protein-ligand complexes: (i) sequence identity of the proteins, (ii) pocket-level Jaccard similarity using pharmacophores, (iii) interaction-level similarity using PLIP features, and (iv) ligand similarities using the Tanimoto coefficient between ECFP4 fingerprints. The algorithm then identifies clusters of similar protein-ligand systems. Finally, the test set is constructed to contain systems from clusters that have no or minimal similarity to any systems in the training or validation set. Along with this, there are two simpler splits: PLINDER-TIME, which is a time-based split, and PLINDER-ECOD, which is based on ECOD topologies.

In Supplementary Table 2 of the original publication, we already showed this table comparing DataSAIL’s splits to PLINDER. This was added on the suggestion of a reviewer. We include it here in Table 3 for completeness of this addendum. Similarly to the LP-PDBBind dataset, the DataSAIL S2 split provides a reduction of data leakage compared to the PLINDER split.

**Table 3:** DataSAIL on PLINDER

Split	Scaled *L*(*π*)
PLINDER-PL50	0.0678
PLINDER-ECOD	0.3601
PLINDER-TIME	0.3682
DataSAIL Ligand S1	0.2307
DataSAIL Protein S1	0.4008
DataSAIL S2	**0.0252**

Comparison of the scaled *L*(*π*) of the different splits of the PLINDER-NR dataset to three DataSAIL splits. The minimal data leakage is highlighted in **bold**.

## PINDER

The Protein INteraction Dataset and Evaluation Resource (PINDER) contains curated and well-annotated PPIs obtained from the RCSB NextGen database^8^. After data cleaning and preprocessing, PINDER provides a split with minimized data leakage. To measure the leakage between two systems (interacting protein-protein pairs), the authors employed FoldSeek^9^ and MMseqs^10^ to compare and cluster protein sequences and structures. In Table 4, we compare DataSAIL to version 1 of PINDER, released in November 2023.

**Table 4:** DataSAIL on PINDER

Split	Scaled *L*(*π*)
PINDER	**0.0068**
DataSAIL	0.0140

Comparison of the scaled *L*(*π*) of the main PINDER split to the described DataSAIL split. The minimal data leakage is highlighted in **bold**.

Other than for the LP-PDBBind dataset, we can define a similarity metric that incorporates both dimensions in this two-dimensional dataset of interacting proteins. Therefore, we did not directly use DataSAIL’s S2 splitting module but rather the S1 with all protein sequences from both dimensions, weighted with the number of interactions each protein participates in. From the resulting assignment, we assigned an interaction to a split if and only if both proteins are assigned to that same split. Here, the PINDER split exhibits less data leakage than the DataSAIL split.

## “Gold standard” human PPI dataset

In their work, Bernett et al.^5^ demonstrate that the performance of many sequence-based models for PPI prediction relies solely on a bias in the data, and that the models memorize the data rather than generalizing from it. Therefore, most models only work because of information leakage, rather than because they accurately understand biology. The authors also provide a dataset to facilitate the evaluation of future models. This dataset is called the “gold standard dataset”^5^ (Table 5).

**Table 5:** DataSAIL on the “gold standard” PPI dataset

Split	Scaled *L*(*π*) Gold
standard dataset	0.3642
DataSAIL	**0.0465**

Comparison of the scaled *L*(*π*) of the provided split by Bernett et al. to the described DataSAIL split. The minimal data leakage is highlighted in **bold**.

To produce it, the authors used KaHIP^11^ to split the whole human proteome into three partitions, *P*_0_*, P*_1_, and *P*_2_. This was done using the all-against-all protein sequence similarity matrix obtained from SIMAP2^12^ with length-normalized bitscores. Based on these partitions, the PPIs from the HIPPIE database v2.3^13^ were assigned to the three partitions if and only if both interacting proteins were in the respective partition. Negative interactions were sampled randomly while preserving the node degree distribution of the respective partition. Lastly, CD-HIT was employed to enforce a sequence similarity threshold of 40% and to remove sequence redundancy. The final dataset has 274,500 interactions.

With DataSAIL, we followed the same procedure as for splitting the PINDER dataset, preserving the split ratios and utilizing stratification to maintain a balance between positive and negative interactions.

## Data Availability

The recommended splits for MoleculeNet were extracted from the Python Package DeepChem v2.8.0. We downloaded the LP-PDBBind dataset from the GitHub Repository at https://github.com/THGLab/LP-PDBBind/blob/master/dataset/LP_PDBBind.csv. For PLINDER and PINDER, we accessed the splits in their respective Google Cloud Storage accounts. For PLINDER, we downloaded the splits for v0 from https://console.cloud.google.com/storage/browser/plinder/2024-04/v0/splits and the data from https://console.cloud.google.com/storage/browser/plinder/2024-04/v1. For PINDER, we downloaded the index.csv.gz from https://console.cloud.google.com/storage/browser/pinder/2023-11. Lastly, we downloaded the “gold standard” dataset from Figshare (10.6084/m9.figshare.21591618.v4). All DataSAIL splits were computed using DataSAIL v1.2.1 and uploaded to Zenodo^14^.
