# Supplementary material for: Data splitting to avoid information leakage with DataSAIL
Source: Nat Commun. 2025 Apr 8;16:3337. doi: 10.1038/s41467-025-58606-8 (PMC11978981; doi:10.1038/s41467-025-58606-8)
Supplement: Supplementary file 1 — Supplementary Information [file 41467_2025_58606_MOESM1_ESM.pdf]

Data splitting to avoid information leakage with DataSAIL

Supplementary information

S1 Detailed description of related work

Common machine learning frameworks such as scikit-learn [1], Therapeutics Data Commons [2], and DeepChem [3] provide data splitting methods, some of which directly work on biochemical data. Moreover, some tools focus on information leakage reduction for biological data, such as LoHi [4], GraphPart [5], and astartes [6]. Importantly, none of these tools can simultaneously split two-dimensional data, considering both dimensions. They offer a random or one-dimensional split for two-dimensional data where information is still leaked along the other dimension. Evaluating models on two-dimensional splits still involves the necessity to compute the splits manually [7, 8]. Some frameworks offer stratified splits, but no tool combines stratification with information leakage minimization. Some tools, such as LoHi and GraphPart, optimize their splits towards less information leakage by deleting data that violate constraints. No tool works for more than one type of biomolecular data. While most splitting mechanisms can be extended to other data types, all tools are limited to small molecules or proteins when used out-of-the-box (see Section 2.1 for details).

In the following, we more extensively discuss other data splitting tools mentioned in the introduction. Tools for splitting biological data can be divided into two groups: tools relying on formulations of the data splitting task as an optimization problem (LoHi, GraphPart) and tools implementing purely procedural algorithms (Therapeutics Data Commons, sklearn, DeepChem). Among tools falling into the second category, DeepChem offers a superset of the data splitting algorithms provided by sklearn and Therapeutics Data Commons. For this reason, sklearn and Therapeutics Data Commons are not covered by the descriptions provided below.

S1.1 DeepChem

DeepChem [3] provides five data splitting modes, which are described below. The major improvements of DataSAIL over the five DeepChem splitting modes are the applicability of DataSAIL to two-dimensional data and the incorporation of stratification into similarity-based splits. Additionally, DataSAIL can split into arbitrarily many blocks at once, while DeepChem only allows to split into three parts.

DeepChem – Butina

This mode is based on the clustering algorithm by Butina [9]. After computing clusters with the Butina algorithm, they are assigned into blocks starting from the largest clusters. This does not take into account inter-cluster similarities.

DeepChem – Fingerprint

In this mode, data can only be split into two blocks simultaneously. DeepChem computes all pairwise Tanimoto similarities between the fingerprints of the input molecules and assigns a random molecule to the first split. The remaining molecules are iteratively assigned by calculating the most dissimilar molecule to one of the two blocks and assigning it to the other. Additionally, this mode can split a dataset into only two blocks simultaneously. Splits with more blocks are computed by iteratively splitting. This may lead to suboptimal results when done multiple times to split a dataset into more parts.

DeepChem – MaxMin

The MaxMin splitting is an application of the Maximum Minimum Diversity Problem described in Porumbel et al. [10]. This algorithm maximizes the minimum diversity between data points in different blocks of a split, e. g., molecules in different blocks.

DeepChem – Scaffold

In this procedure, the molecules are first clustered based on their Bemis-Murcko scaffolds [11]. Then, the clusters are assigned to blocks, starting with the largest, as in the Butina splitting. Similarly, this mode does not take inter-cluster similarities into account.

DeepChem – Weight

Splitting by weight in DeepChem works similarly to Scaffold and Butina splitting. The molecules are sorted by molecular weight from heavy to light and assigned to the blocks in that order. Therefore, all heavy molecules are in the first split, and all light molecules are in the last split. This mode distributes molecules by weight but may assign highly dissimilar molecules with similar weights to the same blocks.

S1.2 LoHi Splitter

The LoHi splitter [4] was developed with two ML tasks on small molecules in mind: (i) lead optimization (Lo) and (ii) hit identification (Hi). Here, we only use the Hi splitting algorithm that defines ILPs to compute data splits. The basis is a graph with nodes for all molecules (or clusters) in the dataset. Edges between them represent a pairwise similarity above a user-defined threshold. Based on this graph, the algorithm constructs an ILP to solve the balanced vertex minimum k-cut problem. The idea is to remove as few nodes as possible to assign the resulting connected components into the blocks matching the size constraints. A downside of this is that the algorithm does not always find a solution, and the user needs biological knowledge to define meaningful thresholds. The tool works only for small chemical molecules, even though its algorithmic approach could easily be extended to other data types.

The major improvements of DataSAIL over the LoHi splitter are the applicability of DataSAIL to two-dimensional data, the incorporation of stratification into the splits, and the fact that DataSAIL does not delete data when used to split one-dimensional data. Furthermore, DataSAIL produces data splits for large datasets reliably (see Figure 6d in the main article). Lastly, the underlying optimization problems are different. We use the minimum k-section problem, and LoHi uses the balanced vertex minimum k-cut problem. The main difference is that in the k-section problem, edges are removed, while in the k-cut problem, vertices, i.e., data points, are removed.

S1.3 GraphPart

Unlike the LoHi splitter, GraphPart [5] is implemented to split DNA, RNA, and protein sequences. It uses an iterative algorithm that optimizes initial clustering based on MMseqs2 or the Needleman-Wunsch algorithm.

The major improvements of DataSAIL over the GraphPart are the applicability of DataSAIL to molecular and nucleic sequence data, two-dimensional data, the incorporation of stratification into the splits, and the fact that DataSAIL does not delete data when used to split one-dimensional data.

S2 Additional results for one-dimensional data

In addition to comparing against a random baseline reported above, we benchmarked DataSAIL's effectiveness in removing similarity-induced information leakage on one-dimensional molecular data against LoHi and several data-splitting algorithms available in DeepChem `v2.7.1`. For this, we trained five of the models discussed in Section 4.5 (except DeepDTA) on datasets from the MoleculeNet collection. MoleculeNet is a good benchmark suite for this because it offers many different datasets of different sizes with different types of molecules. From the original collection, we left out three datasets: (i) QM7b because it is not widely used and the Python package DeepChem does not offer a downloader for it, (ii) PCBA because it is too big with $> 300,000$ molecules, and (iii) PDBBind because it is a protein-ligand interaction dataset.

In Supplementary Figure 1, we see that, on 10 out of 14 datasets, DataSAIL computes splits with the lowest leakage $L(\pi)$, followed by LoHi, which yields $L(\pi)$-minimal splits on 8 datasets (ties on 4 datasets). Furthermore, the ML models are not equally sensitive to information leakage. This is visualized in Supplementary Figure 2, where we correlate the increase/decrease in the four performance measures used in MoleculeNet to the change in the scaled information leakage $L(\pi)$. All changes are relative to the random baseline I1.

For the deep learning model D-MPNN, we additionally compared test performances obtained for DataSAIL's S1 splits to the test performances reported in the original publication [12] (Supplementary Table 1), where the authors had evaluated D-MPNN not only on random splits but also on customized scaffold-based splits to test D-MPNN's OOD generalization capacity (Supplementary Table S4 and S5 in [12]). Overall, DataSAIL's S1 splits yield similarly hard generalization problems as the scaffold splits constructed by the authors of D-MPNN, indicating that out-of-the-box data splitting with DataSAIL can realistically represent relevant OOD scenarios.

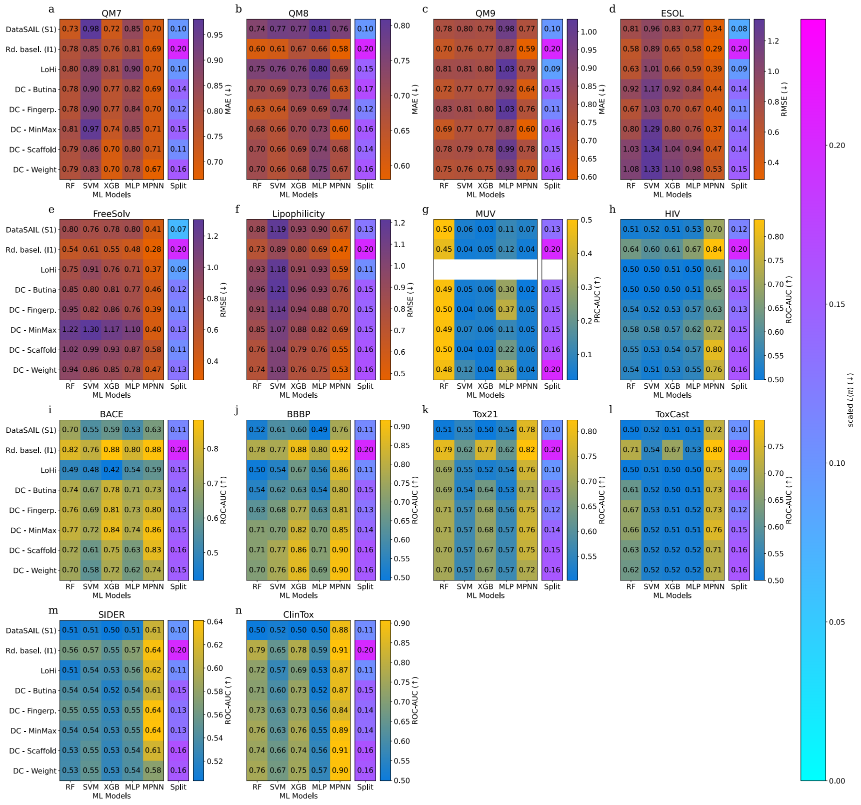

Supplementary Figure 1: **Extensive comparison of splitting algorithms for molecular data.** DataSAIL splits are compared to a random baseline (I1), the LoHi splitter, and the five splits by DeepChem. The color schemes are heatmap-specific, i. e., the colors cannot be transferred between heatmaps. We use two color schemes: Orange-purple to show results on regression datasets (panels **a-f**) and blue-yellow for classification settings (panels **g-n**). LoHi did not produce a split for the MUV dataset. The label "Rd. basel." used in the plots abbreviates "Random baseline". ROC-AUC is the area under the receiver operating characteristic, and PRC-AUC is the area under the precision-recall curve.

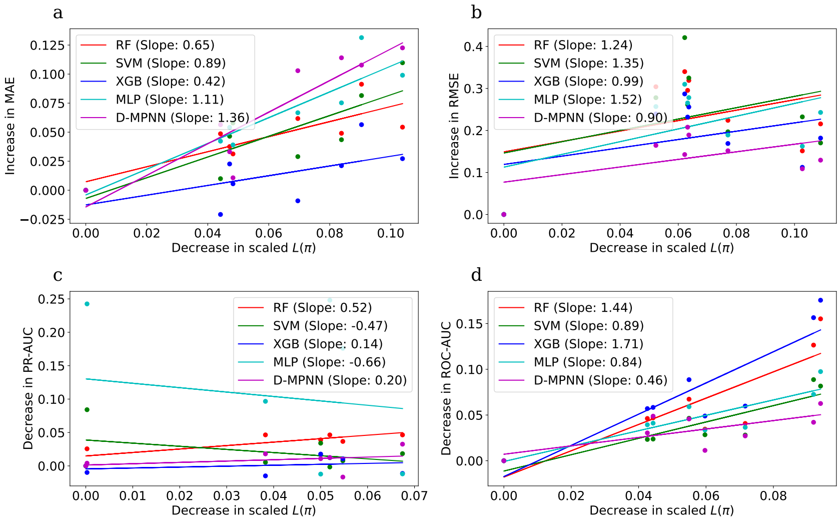

Supplementary Figure 2: **Visualization of the correlation between performance and leakage.** Shown are changes in performance and changes in scaled $L(\pi)$ for the MoleculeNet datasets grouped by performance measure. Panel **a** shows the quantum mechanics datasets QM7, QM8, and QM9. Panel **b** groups ESOL, FreeSolv, and Lipophilicity, the physical chemistry datasets. Panel **c** depicts MUV, and panel **d** BACE, BBBP, Tox21, ToxCast, SIDER, and ClinTox.

S3 Additional results for two-dimensional data

As mentioned in the main article, we are unaware of any tool that can automatically compute data splits of two-dimensional data. However, most tools can compute S1 splits of two-dimensional biological data. To compare against these tools, we again used the LP-PDBBind dataset. We evaluated how DataSAIL performs compared to tools that can split along the drug axis (LoHi and different variants of DeepChem) and a tool that can split along the protein axis (GraphPart).

Supplementary Figure 3 shows the results for tools that compute S1 splits along the drug axis. While some tools succeed in reducing information leakage slightly more than S1 splits computed by DataSAIL, they are all clearly outperformed by DataSAIL's S2 splits. The results for splitting along the protein axis are similar (Supplementary Figure 4): The existing tool GraphPart reduces information leakage slightly more than DataSAIL's S1 splits but is clearly outperformed by DataSAIL's S2 splits. Overall, these results show that when splitting two-dimensional data, simultaneously considering similarities along both dimensions is crucial for obtaining data splits that yield performance estimates that realistically reflect ML model performance during inference in application scenarios where both the proteins and the ligands are OOD in comparison to the training data.

Supplementary Table 1: Test performances of D-MPNN. Comparison of performances on classification datasets reported in the original publication [12] and corresponding performance estimates obtained upon S1 splitting with DataSAIL. For the MUV dataset, we report the area under the precision-recall-curve (PRC-AUC). For all others, the area under the receiver operating characteristic curve (ROC-AUC). Higher values indicate better model performance; best values are marked in bold.

Split	MUV	HIV	BACE	BBBP	Tox21	ToxCast	SIDER	ClinTox
	D-MPNN performances reported in the original publication [12]							
Random	0.12	0.82	0.88	0.91	0.85	0.74	0.65	0.89
Scaffold-based	**0.04**	0.81	0.87	0.89	0.79	**0.69**	**0.61**	**0.82**
	Our results							
DataSAIL (S1)	0.07	**0.70**	**0.63**	**0.76**	**0.78**	0.72	**0.61**	0.88

Supplementary Table 2: PLINDER evaluation. Comparison of scaled $L(\pi)$ on different splits of the PLINDER-NR dataset.

Split	scaled $L(\pi)$
PLINDER-PL50	0.0678
PLINDER-ECOD	0.3601
PLINDER-TIME	0.3682
DataSAIL Ligand S1	0.2307
DataSAIL Protein S1	0.4008
DataSAIL S2	**0.0252**

Recently, the PLINDER benchmark dataset was published to enable leakage-reduced evaluation of ML models to predict protein-ligand interactions [13]. It contains three leakage-reduced splits of protein-ligand interactions: PLINDER-PL50, PLINDER-TIME, and PLINDER-ECOD. The basis of all three splits is the PLINDER-NR dataset, which contains protein-ligand interactions obtained from the Protein Data Bank (PDB) [14] with unique combinations of PDB and Chemical Component Dictionary [15] codes. PLINDER-PL50 splits interactions based on similarity of protein pockets, PLINDER-TIME groups them based on their dates of submission to PDB, and PLINDER-ECOD splits them based on their their pocket-level ECOD ("Evolutionary Classification of Protein Domains") [16] topology annotations.

We used DataSAIL to compute S1 and S2 splits of the PLINDER-NR dataset. In Supplementary Table 2, we can see how the different splitting techniques influence $L(\pi)$. Overall, the S2 split results in the lowest data leakage, closely followed by PLINDER-PL50. Although DataSAIL is a general-purpose tool, it hence yields a split with less data leakage than the PLINDER-PL50 split, which was specifically constructed for the PLINDER-NR dataset. Both splits remove data from the dataset

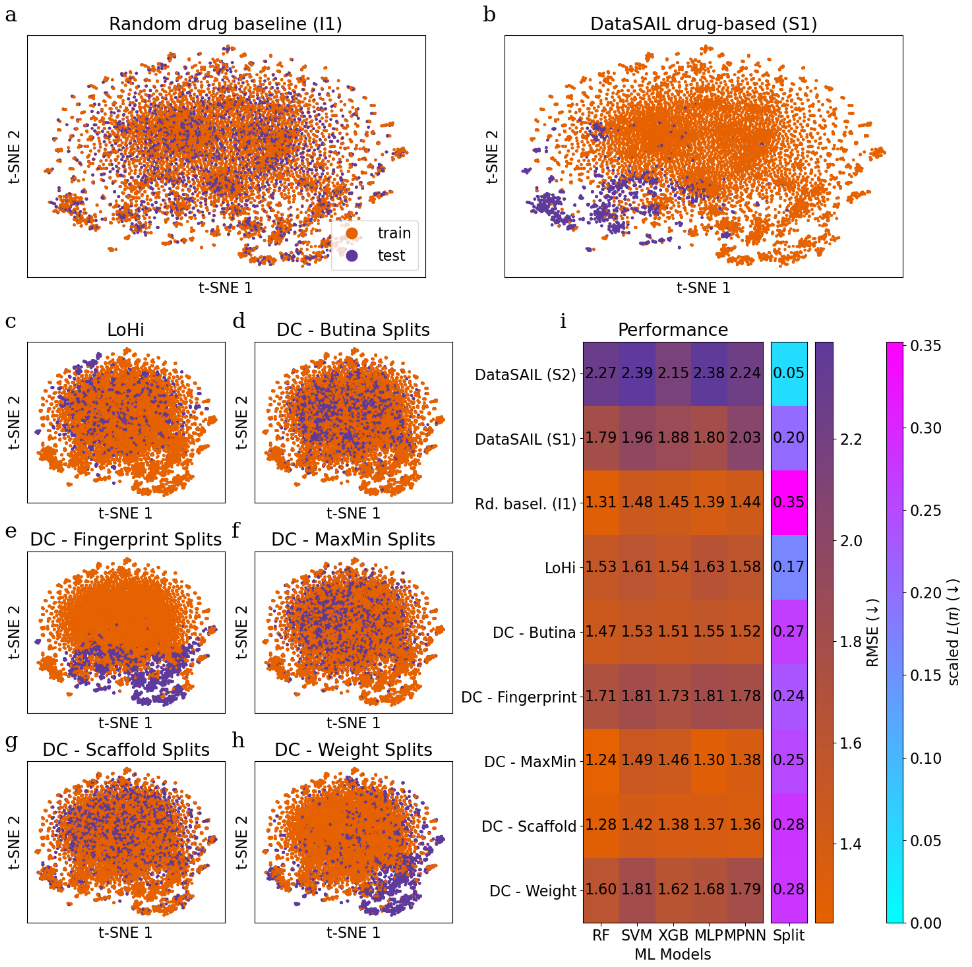

Supplementary Figure 3: **Extensive comparison of splitting algorithms on ligand data from LP-PDBBind.** This analysis examines the t-SNE embeddings produced by various splitting methods applied to LP-PDBBind drugs (panels **a-h**). Panel **i** compares the eight ligand-based splits against DataSAIL's two-dimensional split, S2.

(around 50,000 interactions for DataSAIL's S2 split and around 36,000 interactions for PLINDER-PL50).

Supplementary References

[1] Pedregosa F., Varoquaux G., Gramfort A., Michel V., Thirion B., Grisel O., Blondel M., Prettenhofer P., Weiss R., Dubourg V., Vanderplas, V., Passos, A.,

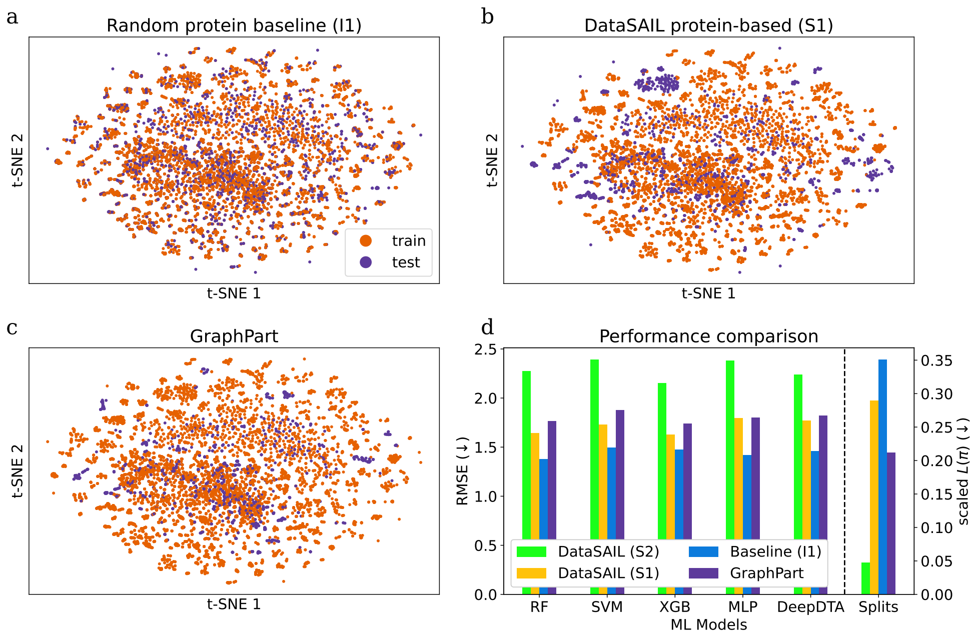

Supplementary Figure 4: Extensive comparison of splitting algorithms on protein data from LP-PDBBind. We compare the latent space embeddings generated by various protein-dimension splitting methods applied to LP-PDBBind data (panels **a-c**). Panel **d** highlights the performance of three protein-based splits in contrast to DataSAIL's two-dimensional split, S2.

Cournapeau, D., Brucher, M., Perrot, M., Duchesnay, É. Scikit-learn: Machine Learning in Python. *Journal of Machine Learning Research* **12**, 2825–2830 (2011).

[2] Huang K., Fu T., Gao W., Zhao Y., Roohani Y, Leskovec J., Coley C. W., Xiao C., Sun J., Zitnik M. Artificial intelligence foundation for therapeutic science. *Nature Chemical Biology* **18**, 1033–1036 (2022).

[3] Ramsundar B., Eastman P., Walters P., Pande V., Leswing K., Wu Z. Deep Learning For The Life Sciences (O'Reilly Media, Sebastopol, CA, 2019)

[4] Steshin S. Lo-Hi: Practical ML Drug Discovery Benchmark. Preprint at https://arXiv.org/abs/2310.06399 (2023).

[5] Teufel F., Gíslason M. H., Almagro Armenteros J. J., Johansen A. R., Winther O., Nielsen H. GraphPart: homology partitioning for biological sequence analysis. *NAR Genomics and Bioinformatics* **5**, lqad088 (2023).

[6] Burns J. W., Spiekermann K. A., Bhattacharjee H., Vlachos D. G., Green W. H. Machine Learning Validation via Rational Dataset Sampling with astartes. *Journal of Open Source Software*, **8**, 5996 (2023).

[7] Vall A., Hochreiter S., Klambauer G. BioassayCLR: Prediction of biological activity for novel bioassays based on rich textual descriptions. *ELLIS Machine Learning for Molecule Discovery Workshop* (2021). Available at https://www.bioinf.jku.at/people/klambauer/02.pdf.

[8] Götz J., Jackl M. K., Jindakun C., Marziale, A. N., André, J., Gosling, D. J., Springer, C., Palmieri, M., Reck, M., Luneau, A., Brocklehurst, C. E., Bode, J. W. High-throughput synthesis provides data for predicting molecular properties and reaction success. *Science advances* **9**, eadj2314 (2023).

[9] Butina D. Unsupervised data base clustering based on daylight's fingerprint and Tanimoto similarity. *Journal of Chemical Information and Computer Sciences* **39**, 747–750 (1999).

[10] Porumbel D. C., Hao J.-K., Glover F. A simple and effective algorithm for the MaxMin diversity problem. *Annals of Operations Research* **186**, 275–293 (2011).

[11] Bemis G. W., Murcko M. A. The properties of known drugs. 1. Molecular frameworks. *Journal of medicinal chemistry* **39** 2887–2893 (1996).

[12] Yang K., Swanson K., Jin W., Coley C., Eiden, P., Gao, H., Guzman-Perez, A., Hopper, T., Kelley, B., Mathea, M., Palmer, A., Settels, V., Jaakkola, T., Jensen, K., Barzilay, R. Analyzing learned molecular representations for property prediction. *Journal of Chemical Information Modeling* **59**, 3370–3388 (2019).

[13] Durairaj J., Adeshina Y., Cao Z., Zhang X., Oleinikovas V., Duignan T., McClure, Z., Robin, X., Kovtun, D., Rossi, E., Zhou, G., Veccham, S., Isert, C., Peng, X., Sundareson, P., Akdel, M., Corso, G., Stärk, H., Carpenter, Z., Bronstein, M., Kucukbenli, E., Schwede, T., Naef, L. PLINDER: The protein-ligand interactions dataset and evaluation resource. Preprint at https://www.biorxiv.org/content/10.1101/2024.07.17.603955 (2024).

[14] Berman H. M., Westbrook J., Feng Z., Gilliland G., Bhat, T. N., Weissig, H., Shindyalov, I. N., Bourne, P. E. The Protein Data Bank. *Nucleic Acids Research* **28**, 235–242 (2000).

[15] Westbrook J. D., Shao C., Feng Z., Zhuravleva M. Velankar S., Young J. The chemical component dictionary. *Bioinformatics* **31**, 1274–1278 (2015).

[16] Cheng H., Schaeffer R. D., Liao Y., Kinch L. N., Pei, J., Shi, S., Kim, B.-H., Grishin, N. V. ECOD: an evolutionary classification of protein domains. *PLoS Computational Biology* **10**, e1003926 (2014).
